# Thymoquinone: From *Nigella sativa *to a protective pharmacological compound in managing opioid dependence and amphetamine type stimulant issues 

**DOI:** 10.22038/ijbms.2020.41678.9841

**Published:** 2020-07

**Authors:** Liyana Hazwani Mohd Adnan, Nor Hidayah Abu Bakar, Nordin Simbak, Nasir Mohamad, Rusli Ismail, Nor Zidah Ahmad, Nor Suliana Mustafa, Nurul Farah Aina Md Fauzi

**Affiliations:** 1 Faculty of Medicine, University Sultan Zainal Abidin, City Campus, 20400 Kuala Terengganu, Malaysia

**Keywords:** Amphetamine, Methadone, Morphine toxicity, Opioid dependence – syndromes, Thymoquinone

## Abstract

Opioids, amphetamines, and other types of substances have been widely abused around the world. Opioid dependence and tolerance are two distinct phenomena that have been associated with substance abuse issues. The management of its adverse consequences is becoming more challenging. More and more people are treated in Methadone Maintenance Therapy (MMT) program yet the issues are still unresolved. Researchers are continuing to study the best formulation in treating opioid dependent people starting with modern and alternative drug therapies. Since 2008 , thymoquinone (TQ) has been extensively studied by researchers around the world and has emerged to be a new potential drug candidate in managing substance abuse issues. Thus, the aim of this article is to review the effects that TQ may have on opioid dependent subjects and other abused substances such as amphetamine may have been studied. All of the articles from 2008 until 2019 involving the effects of TQ on substance abuse from Google Scholar®, Scopus®, and Pubmed® databases have been searched and reviewed. The keywords used were thymoquinone, opioid dependence, amphetamine, and Nigella sativa. The research results also have been discussed in this article. Based on the research conducted, TQ was effective in reducing the adverse health consequences associated with substance abuse such as withdrawal symptoms, tolerance, and cell damages. It is concluded that TQ could be a potential drug that can be complemented with the currently available drugs in substance abuse therapies.

## Introduction

Opioid-type drugs are continuously being abused although many adverse health outcomes have been revealed. Methadone maintaining therapy (MMT) remains to be the only registered detoxification treatments available for opioid-dependent people in private and public healthcare institutions in Malaysia. However, many issues regarding methadone treatments have been raised among opioid-dependent individuals. It was claimed that the withdrawal symptoms had become worse and lasted longer due to longer methadone half-life (1). Thus, this had caused drop-out or failure in the MMT program. Scientifically, it also creates a chemical dependency among the opioid-dependent people and some of the subjects were associated with prolonged QTc intervals (2). These issues had raised immediate attention to optimize the MMT program towards reducing the above said problems by supplementing methadone with other alternative drugs. It has been said that in any treatment of any disease, the effectiveness is not 100%, the efficacy is not 100% and even no drugs are 100% safe. For opioid dependence, there have not been so many drugs that are clinically used. Thus, new drugs and new treatments are always welcome. One of the most currently well-known alternative drugs that has been extensively studied in the management of opioid dependence and methadone is thymoquinone (TQ), a pharmacologically active compound isolated from *Nigella sativa* (linn seed) oil. Hence, this article will discuss the therapeutic role of TQ in managing opioid dependence therapy. TQ has been reported for its high therapeutic potential in a number of medical conditions (3), including substance abuse therapy. It also has potential chemical effects mimicking opioids, especially on the mechanisms of opioid dependency and tolerance. It is an opioid receptor stimulating compound with 45% ligand displacement at *mu*-opioid receptor (4), which is a receptor that is critical for morphine’s rewarding effects. In this article, all of the recent outcomes regarding the role of TQ in managing opioid dependence therapy and a few on Amphetamine Type Stimulants (ATS) will be reviewed.

***TQ in opioid dependence therapy***

*N. sativa* has been reported to have many medical properties since ancient times (5). It has been shown to exhibit a number of pharmacological properties including anti-inflammatory, antioxidant, analgesics, immuno-protective, anti-microbial, and anticonvulsant properties (6, 7). Yet, the therapeutic role of *N. sativa* in opioid dependence therapy had not been revealed until 2008. *N. sativa* was first introduced in opioid dependence therapy in 2008 by Sangi *et al*. For centuries, opioid dependence had been a major social and psychiatric problem worldwide. However, it was realized that there were no non-opioid treatments available for opioid dependent people although there were so many herbs and plants that had been used for healing purposes. Since then, Sangi *et al.* have carried out new and novel research to investigate the effects of *N. sativa *in reducing opioid withdrawal syndromes among 35 known opioid addicts. In his study, *N. sativa *500 mg was administered three times daily and it was shown that *N. sativa* 500 mg had reduced the subjective opioid withdrawal symptoms from pre-treatment day-3 scoring rate of 63.2±13.57 to 14.56±8.13 at day 12. Similarly, the objective opioid withdrawal signs were also reduced from pre-treatment day-3 scoring rate of 25.52±3.08 to 7.72±2.35 at day 12. Based on the results, they concluded that non-opioid drug *N. sativa *is effective in long term treatment of opioid dependence (8). 

Since then, the effects of *N. sativa* in opioid dependence therapy became extensively studied. Five years after the study from Sangi *et al.*, researchers began to introduce the effects of a pharmacologically active compound from *N. sativa* seed’s oil such as TQ in opioid dependence therapy. TQ had been vastly studied in numerous medical conditions and found to be effective in treating chronic illnesses such as breast cancer cells (9), depressions, seizures, and many more (10, 11). However, the effects of TQ in opioid tolerance and dependence are inadequate. Thus, a group of research led by Abdel-Zaher investigated the role of TQ in morphine-induced tolerance and dependence in mice by focusing on the brain oxidative stress and inducible nitric oxide synthase expression. They found that co-administration of TQ along with morphine significantly inhibited morphine-induced progressive increase in brain malondialdehyde (MDA) level and nitric oxide (NO) production as well as progressive decrease in brain intracellular reduced glutathione (GSH) level and glutathione peroxidase (GSH-Px) activity. The results from their study contributed to important knowledge in the literature of opioid addiction research showing that TQ significantly inhibits morphine-induced oxidative stress, NO overproduction, and increase in brain-inducible NO synthase expression, hence attenuating the development of morphine tolerance and dependence in mice (12). 

Following the research, in 2014, there was a review on the phytotherapy of opioid dependence and withdrawal syndromes by Tabatabai and team. *N. sativa* had been grouped into the Ranunculaceae plant family. According to the article, among all 35 plant species that had been studied in opioid dependence subjects, plants from Ranunculaceae are among the most effective therapies other than Lamiaceae and Apiaceae plant families (13), showing another evidence for the effectiveness of *N. sativa* in reducing opioid dependence issues. 

Methadone Maintenance Therapy (MMT) is widely used in treating opioid withdrawal syndromes but with limited efficacy. Several issues have been addressed regarding patients on long-term MMT, including poor retention rates (14), worsened withdrawal syndromes due to longer methadone half-life, prolonged QTc interval, and the development of chemical dependency (15, 16). Realizing the extensive attention given to *N. sativa* and its active compound, TQ, in opioid withdrawal studies, TQ has been suggested to be used as a new potential supplement for MMT towards optimizing the output of the MMT program. The basis of this suggestion is believed to be mediated by calcium channel that is in line with the properties of TQ as calcium channel blocker. It was believed that calcium channel blocker had been responsible for attenuating the effects of morphine withdrawal as shown by standard calcium channel blocker drugs such as verapamil and felodipine, which effectively reduce opioid withdrawal syndromes (17).

More studies have been conducted regarding the role of TQ. Recently, a study on the investigation of the effects of TQ in attenuating morphine-induced tolerance and dependence in mice was further being carried out by Hosseinzadeh *et al*. In their research, from 10mg/kg to a maximum of 40 mg/kg TQ injected to the mice, they found that TQ significantly attenuated the withdrawal signs of morphine dependent mice in a dose-dependent manner with dose of 40 mg/kg significantly greater than 20 mg/kg. Interestingly, the effects of 40 mg/kg of TQ were comparable to that of diazepam. The results of this study proved the potential of TQ in preventing the development of morphine-induced tolerance and dependence (18). 

Moreover, the results from the study were further strengthened by the effects of TQ at molecular and cellular levels. At molecular levels, the first attempt was done by a group of researchers to investigate the effects of TQ in opioid dependence by using RNA sequencing technologies with the advent of Next Generation Sequencing (NGS) technology. Interestingly, they found that TQ up-regulated several key genes in the morphine addiction pathway, which comprises phosphodiesterase 1 A (PDE1A) genes, gamma-aminobutyric acid type A receptor theta subunit (GABRQ), and G protein subunit beta 3 (GNG3) genes in which these genes were down-regulated by chronic morphine (19). 

At cellular levels, TQ was significantly proven to attenuate the morphine-induced cyclic Adenosine Monophosphate (cAMP) overshoot via a study on morphine withdrawal using opioid receptor-expressing cell line U87 glioblastoma cells. The effect could be seen after the cells were co-treated with morphine and TQ (17). At cellular levels, opioid withdrawal is defined as a high overshoot of cAMP levels far above the control values and this phenomenon (an adaptation in cAMP pathways) showed that opioid tolerance and dependence could be studied at the single-cell level (20). 

TQ also has many therapeutic properties including anti-inflammatory, antioxidant and immunomodulatory effects. Continuous exposure to morphine has long been associated with apoptotic and oxidative damage to the cells. Surprisingly, co-treatment of TQ with morphine was shown to decrease NO, and total antioxidant capacity (TAC) levels had increased significantly. Interestingly, TQ (9 and 18 mg/kg) plus morphine also caused a significant decrease in mRNA expressions of the genes involved in the apoptotic pathway, which are p53 and Bax if compared with the morphine-treated group, suggesting another protective role of TQ in managing opioid dependence issues (21). The protective role of TQ towards preventing morphine-induced injuries to mice kidneys were also noted. The co-treatment of various doses of TQ (4.5 mg/kg, 9 mg/kg, and 18 mg/kg) with morphine intraperitoneally in mice was shown to significantly decreased the blood urea nitrogen, serum creatinine, and serum NO levels compared with the morphine group. Moreover, the weight of the kidney, the number and the mean diameter of the glomeruli that were reduced in morphine-treated groups were also significantly enhanced by TQ (22).

***TQ in managing amphetamine type stimulant***

The beneficial role of TQ was not only limited to opioid dependence therapy but was extended to the other types of misused substances such as Amphetamine Type Stimulant (ATS). Researchers continue to reveal its potential effects on amphetamine and proposed TQ as a better candidate in managing amphetamine issues due to its interaction with neurotransmitter dopamine (23). It is widely known that amphetamine drugs induce neurotoxicity by causing a dysfunction in several mechanisms, predominantly on dopamine system (24), yet there is still no specific drugs available for amphetamine abusers to reduce their withdrawal symptoms and addiction like methadone that is prescribed for opioid dependent individuals (25–28). 

Another type of illicit drug under the ATS group is 3,4-methylenedioxymethamphetamine (MDMA; “ecstasy”). Recently, the protective role of TQ in the management of MDMA toxicity has been elucidated. The long-term effect of MDMA includes depletion of extracellular serotonin or 5-hydroxytryptamine (5-HT). However, the recent study demonstrated a significant increase in 5-HT levels in the MDMA-TQ group as compared with the MDMA group. Moreover, the MDMA-TQ group also showed a higher percentage of weight gain as compared to the MDMA group. The outcomes from this study then suggested that the long-term MDMA induced 5-HT depletion in rat cerebrospinal fluid could be prevented by TQ (29). 

**Table 1:** Data summary of Nigella sativa and thymoquinone in opioid dependence and ATS issues

Year	Data summary of *** N. sativa ***and thymoquinone	Authors
2008	***N. sativa*** 500 mg reduced the subjective opioid withdrawal symptoms from 63.2 ± 13.57 to 14.56 ± 8.13. Similarly, the objective opioid withdrawal signs were also reduced from the scoring rate of 25.52 ± 3.08 to 7.72 ± 2.35.	Sangi *et al*.
2013	Co-administration of thymoquinone along with morphine significantly inhibited morphine-induced progressive increase in brain MDA level and NO production as well as progressive decrease in brain GSH level and GSH-Px activity.	Abdel-Zaher *et al*.
2014	Plants from the Ranunculaceae family constitute the most effective therapy other than Lamiaceae and Apiaceae plant families in opioid dependent subjects.	Tabatabai *et al.*
2016	Thymoquinone (10 mg/kg- 40 mg/kg) significantly attenuated the withdrawal signs of morphine dependent mice in a dose-dependent manner with a dose of 40 mg/kg significantly greater than 20 mg/kg.	Hosseinzadeh *et al.*
2017	Thymoquinone up-regulated several key genes in the morphine addiction pathway, which are PDE1A, GABRQ, and GNG3 genes.	Adnan *et al.*
2017	The co-treatment of TQ (4.5 mg/kg, 9 mg/kg, and 18 mg/kg) with morphine intraperitoneally in mice significantly decreased the blood urea nitrogen, serum creatinine, and serum nitric oxide levels from mice kidneys.	Jalili *et al.*
2018	TQ was significantly proven to attenuate the morphine-induced cAMP overshoot via a study on morphine withdrawal in U87 glioblastoma cell lines.	Liyana *et al.*
2018	TQ (9 and 18 mg/kg) plus morphine decreased p53 and Bax mRNA expressions	Jalili *et al.*
2018	MDMA-TQ treated group had a significant increase in 5-HT level as compared with the MDMA group	Mustafa *et al.*

## Conclusion

In summary, all of the available data and resources regarding the therapeutic effects of TQ and *N. sativa* in opioid dependence and MDMA issues have been summarised in Table 1. 

Most of the recent data regarding the role of TQ in opioid dependence therapy and ATS demonstrated the effectiveness of TQ in reducing opioid dependence and ATS adverse health consequences. It is urged to do more research regarding the effects of TQ in opioid dependence and ATS issues to include more information in the literature, as the data available are still scarce. Furthermore, the supplementation of TQ in substance abuse therapy also has been suggested for better outcomes in managing substance abuse detoxification treatments and hence contributing to healthy citizens and peaceful communities. 
